# Factors influencing GPs’ perception of specialised palliative homecare (SPHC) importance – results of a cross-sectional study

**DOI:** 10.1186/s12904-020-00603-3

**Published:** 2020-08-03

**Authors:** K. Stichling, M. Krause, B. Ditscheid, M. Hach, M. Jansky, M. Kaufmann, T. Lehmann, W. Meißner, F. Nauck, W. Schneider, S. Schulz, H. C. Vollmar, U. Wedding, J. Bleidorn, A. Freytag, the SAVOIR Study Group, Anna Bauer, Lia Bergmann, Bianka Ditscheid, Cornelia Eichhorn, Antje Freytag, Michaela Hach, Ulrike Hammer, Aicko Helbig, Beata Hennig, Maximiliane Jansky, Michelle Kaufmann, Markus Krause, Sabine Krauss, Thomas Lehmann, Helmut L’hoest, Srikanth Maddela, Ursula Marschall, Martial Mboulla, Winfried Meißner, Heiner Melching, Florian Mühler, Cornelia Nageler, Friedemann Nauck, Judith Rothaug, Joachim Saam, Werner Schneider, Sven Schulz, Kathleen Stichling, Horst C. Vollmar, Julia von Hayek, Ulrich Wedding, Marie-Luise Völker, Vivienne Kley, Jana Feustel, Ketura Herklotz

**Affiliations:** 1 Institute of General Practice and Family Medicine, Jena University Hospital, Jena, Germany; 2 German Working Group for SAPV, Berlin, Germany; 3 Clinic for Palliative Medicine, University Medical Center Göttingen, Göttingen, Germany; 4 Center for Clinical Studies, Jena University Hospital, Jena, Germany; 5 Department of Palliative Care, Jena University Hospital, Jena, Germany; 6 Center for Interdisciplinary Health Research, University of Augsburg, Augsburg, Germany; 7 Institute of General Practice and Family Medicine, Faculty of Medicine, Ruhr University Bochum, Bochum, Germany

**Keywords:** General practice, Primary palliative care, Specialised palliative homecare, Home care service, Surveys and questionnaires, Cross-sectional survey, Policy implications

## Abstract

**Background:**

General Practitioners (GPs) are the main providers of primary palliative care (PPC). At the same time they are the main initiators of specialised palliative homecare (SPHC). In Germany, little is known about factors which influence GPs in their involvement of SPHC. Aim of our study is to identify factors that drive GPs to give value to and involve SPHC.

**Methods:**

A cross-sectional survey was performed. In 2018, questionnaires were mailed to 6000 randomly selected GPs from eight German federal states, focusing on the extent of GPs’ palliative care activities and their involvement of SPHC.

**Results:**

With a response rate of 19.4% and exclusion of GPs working in SPHC-teams, *n* = 1026 questionnaires were appropriate for analysis. GPs valued SPHC support as the most “important/very important” for both “technical/invasive treatment measures” (95%) and availability outside practice opening hours (92%).

The most relevant factor influencing perceived SPHC-importance was GPs’ self-reported extent of engagement in palliative care (β = − 0.283; CI 95% = − 0.384;−0.182), followed by the perceived quality of utilised SPHC (β = 0.119; CI 95% = 0.048;0.190), involvement in treatment of palliative patients after SPHC initiation (β = 0.088; CI 95% = 0.042;0.134), and conviction that palliative care should be a central part of GPs’ work (β = − 0.062; CI 95% = − 0.116;−0.008). Perceived SPHC-importance is also associated with SPHC-referrals (β =0.138; *p* < 0.001). The lower the engagement of GPs in palliative care, the more they involve SPHC and vice versa.

**Conclusions:**

GPs with low reported activity in palliative care are more likely to initialise SPHC for palliative care activities they do not deliver themselves for various reasons, which might mean that the involvement of SPHC is substitutive instead of complementary to primary palliative care. This finding and its interpretation should be given more attention in the future policy framework for (specialised) palliative homecare.

**Trial registration:**

German Clinical Trials Register DRKS00014726, 14.05.2018.

## Background

In Germany, similar to other high-income countries, specialised palliative homecare (SPHC) has evolved during recent decades to deliver homecare for patients with particularly complex palliative needs. As SPHC demands more resources than primary palliative care (PPC, Table 1), according to the Social Health Insurance legislation [3] (§1 Abs. 6), SPHC can be referred to if there is medical need for specialist palliative care and PPC alone is not sufficient. Thus, SPHC provides complementary care to enable homecare until the end of life even for patients in complex situations (Table 1).

**Table 1:** Two kinds of palliative homecare in Germany

**PPC** ➣ Palliative homecare is mostly delivered as primary palliative care (PPC) [1]). ➣ PPC provided by General Practitioners (GPs) is often supported by nursing care services, ambulatory hospice services, etc. ➣ About 90% of patients at end of life can be sufficiently cared for with PPC, according to estimations of the German Association of Palliative Medicine [2].	**SPHC** ➣ Specialised palliative homecare (SPHC) for patients with severe, advanced, life-limiting illnesses and complex symptoms is funded by the Social Health Insurance. ➣is provided by multi-professional teams (physicians, nurses, other professionals), delivering specialised palliative care at patients’ homes/ nursing homes etc. [3]. ➣ comprises care which in kind, severity and complexity can only be delivered by physicians with extra palliative qualifications, as well as 24-h availability. ➣ requires a referral from GPs, primary care specialists or upon discharge from hospital. ➣ main indications are complex, pronounced symptoms such as neurological, psychiatric or psychological, respiratory or cardiac symptoms, ulcerating wounds or tumours.

Since the introduction of SPHC in 2007, the number of SPHC referrals has continued to rise [4], though with strong regional variations [2].

While GPs are the main providers who take responsibility for outpatient palliative care activities (most of them within PPC but some of them as additionally qualified physicians in SPHC teams), they are at the same time the main initiators and refer to SPHC [5]. This crucial role has not yet been explored sufficiently [6]. We know some of the factors driving GPs’ involvement in palliative care [7, 8] as well as hindering and promoting factors for cooperation between GPs and specialist palliative care teams [9]. There are findings, that GPs lack confidence and skill in identifying and caring for complex patients and time for professional development in palliative care [10]. Working in smaller GP practices, having limited current involvement in palliative care provision, and having less experience or education in palliative care were identified as factors facilitating British GPs in handing over to specialists by a qualitative study [11].

However, we lack reliable evidence about the factors that drive GPs to involve SPHC, which could provide insight into the interrelation of PPC and SPHC. Such evidence is required for further developing the policy framework for both SPHC and PPC, in order to promote access, effectiveness and efficiency for patients in need of palliative care.

To explore the factors that determine the valuing of and involvement in SPHC on behalf of GPs, we conducted a written survey of 6000 GPs in eight federal states in Germany.

This study is part of the collaborative research project SAVOIR (evaluation of specialised palliative homecare (SPHC) in Germany: outcomes, interactions, regional differences) [12].

## Methods

### Hypothesis/Theory

We hypothesised that there are more factors implicated than medical indication of patients’ special need when German GPs involve SPHC. From this survey, we expected insight into a large set of physician-related, practice-related, environmental factors, and in particular, the GPs’ own provision of general palliative care activities as potential factors driving GPs to involve SPHC.

### Study design

We performed a cross-sectional study among GPs in eight German federal states. The data were generated from a postal questionnaire.

### Survey setting and sample

In Germany, the regular delivery of primary health care is organised by 17 Associations of Statutory Health Insurance Physicians (“Kassenärztliche Vereinigung”) corresponding to the federal states.

From the 17 Associations, eight were selected for their greatest heterogeneity regarding structures and policy framework in SPHC (different forms delivery contracts, ownership, team structure, remuneration systems), as well as the number of inhabitants and size. A randomly selected sample of 6000 GPs within these federal states (*n* = 750 each) were invited to participate.

### Instrument

The development of the six-page questionnaire is explained in Additional file 1.

The questionnaire (Additional file 2) comprised of more than 100 items focusing on physician-related characteristics, such as experience with and qualifications in palliative care, self-assessed competence and role in palliative care, workload and practice-related factors, as well as SPHC referral practice, and availability/quality of care of the surrounding palliative care infrastructure. In particular, GPs were asked how often they perform activities for their palliative patients out of the following five categories: assessment and planning of care; symptom management; interventions; coordination of care; and availability. In addition, GPs assessed their need for support from palliative specialists for each activity.

### Data collection and consent of participants

The survey was undertaken from March to May 2018. All questionnaires were accompanied by a personalised letter and a self-addressed stamped envelope. Reminders were sent to all 6000 GPs after four and six weeks, respectively. Informed consent was implied if the completed questionnaire was sent back. After data collection, questionnaires were transcribed electronically into a SPSS Statistics database. Missing values were defined in advance. To ensure the quality of data, a 100% full check of the questionnaires was conducted, revealing no substantial mismatch between the imported data and the original questionnaires.

### Data analysis

Statistical analyses were performed using SPSS Version 25 for Windows.

For valued SPHC support, an additional variable, “SPHC importance index”, was calculated from the average of all sub-questions about palliative activities. With this index, the intensity to which respondents valued SPHC support was qualified on a continuous scale with a minimum score of 1 (“not required”) and a maximum score of 3 (“very important”). For the aggregation of GPs’ extent that they are engaged in palliative care delivery, the “GPs palliative care activity index” was calculated from the average of all sub-questions about palliative activities delivered by GPs from the assessed range from 1 = never to 4 = always.

To analyse which factors were associated with GPs’ perception of the importance of SPHC, we performed univariate and multiple linear regression using all independent variables with the new variable SPHC importance index as the dependent variable. All characteristics were simultaneously entered into the model to calculate the independent effects. The results were considered statistically significant if p ≤ 0.05.

## Results

Reporting refers to the internationally accepted standards of good practice in reporting of survey research (i.e. STROBE) [13, 14].

### Characteristics of respondents

The response rate was 19.4% (*n* = 1166). 1144 (19.1%) questionnaires were eligible. GPs working as a part of a SPHC team (*n* = 118; 10.3%) were excluded from further analysis due to possible conflict of interest. This left a number of *n* = 1026 questionnaires to be analysed.

Table 2 shows the characteristics of the respondents. About half of the GPs were female (50.8%). The average age was 54.1 years, 49.8% worked in a single-handed practice, 45.6% in a group practice and a smaller number in a medical care centre (4.6%). GPs cared, on average, for 18.4 palliative patients (SD = 26.8; Median = 10, IQR = 14) over the past year. The vast majority of the respondents (83.7%) had already referred to SPHC.

**Table 2:** Characteristics of respondents and practice of SPHC referrals^a^

Characteristics	Value			
Respondents - n	1026			
Age - years Mean ± SD	54.1 ± 9.4			
Gender - n (%)				
Male	502 (49.0)			
Female	520 (50.8)			
Work experience in the outpatient sector - years Mean ± SD	20.2 ± 11.2			
Working hours/week - Mean ± SD	46.9 ± 12.4			
Practice type - n (%)				
Single-handed	510 (49.8)			
Group practice	467 (45.6)			
Medical care centre	47 (4.6)			
Employed - n (%)				
Yes	123 (12.0)			
No	887 (86.6)			
Location of practice - n (%)				
Rural (≤ 5,000 inhabitants)	264 (25.8)			
Small town (> 5,000–20,000 inhabitants)	290 (28.3)			
Medium-sized town (> 20,000–100,000 inhabitants)	198 (19.3)			
Big city (> 100,000 inhabitants)	264 (25.8)			
Number of patients/quarter - Mean ± SD	1130.3 ± 373.3			
Number of palliative patients/year - Mean ± SD	18.4 ± 26.8			
Number of home visits/week - Mean ± SD	15.3 ± 20.6			
Number of home visits/quarter within palliative patients - Mean ± SD	15.1 ± 25.5			
**GPs’ attitude towards palliative care**				
	bad	…	…	good
Please assess your overall palliative competence/expertise! - n (%)	20 (2.0)	90 (8.8)	533 (52.1)	350 (34.2)
	not true	rather not true	rather true	completly true
Care of severe sick and dying patients should be a central part of GPs work. - n (%)	34 (3.3)	106 (10.4)	219 (21.4)	657 (64.2)
**SPHC referral practice**				
Have you ever referred SPHC (within your work as a GP)?				
Yes - n (%)	847 (83.7)			
No - n (%)	163 (15.9)			
Number of GPs’ SPHC referrals (follow-up referrals included) - Mean ± SD	8.8 ± 11.9			
Number of SPHC referrals by other health care professionals - Mean ± SD	3.7 ± 5.2			
	never	…	…	always
Do you regularly stay involved in palliative patients’ treatment after SPHC initiation? - n (%)	53 (5.2)	174 (17.0)	298 (29.1)	356 (34.8)
How often is one of your SPHC referrals denied (by MDK)? - n (%)	653 (63.8)	166 (16.2)	31 (3.0)	0

^a^ Not all respondents answered every question. Percent does not add up to 100 due to rounding and non-responses

^b^ Medical advisary service of Social Health Insurance (MDK: Medizinischer Dienst der Krankenkassen)

For non-responder analysis, we performed a statistical comparison between the characteristics of participating GPs and average German GPs [15]. Respondents were more likely to be female (50.8% vs 46.3%) and have additional qualifications in palliative care (9.7% vs 3.5%). Older GPs (> 65 years) were less likely to participate in our survey (9.9% vs 16.3%).

### GPs’ evaluation of SPHC importance

In total, the SPHC importance index averaged 2.25 (SD = 0.51, *n* = 956, min = 0, max = 3) on a continuous scale with 1 “not required”, 2 “important” and 3 “very important” reflecting an overall high rating of the importance of SPHC services by GPs. However, results for single activities for which GPs consider the support of a specialised palliative care team beneficial (Additional file 3) reveal substantial variations: “Technical and invasive treatment measures” was the activity where support was most frequently deemed beneficial by GPs (73% “very important” and 22% “important”). SPHC support in “treatment in the final phase” was also valued as “very important” (59%) or “important” (32%) . For providing 24-h availability to the patient and relatives in the form of home visits outside practice opening hours, support by SPHC was rated “very important” (66%) or “important” (25%). SPHC support through telephone advice outside practice opening hours was similarly rated as “very important” (65%) or “important” (25%).

Approximately 44% of the GPs declared no need for support in terms of “advice/assistance in advance directive and power of attorney for personal care” and “treatment of chronic diseases” (41%) which were the highest scores for “not required”.

### Factors influencing GPs’ perception of the importance of SPHC

In univariate analyses (Table 3), variables which contributed to a higher SPHC importance index were (descending order according to regression coefficient): perceived quality of utilised SPHC services, gender (female), and quality of surrounding palliative infrastructure. Additionally, if GPs felt regularly involved in the treatment of their patients by the SPHC team, they valued SPHC services as being more important.

**Table 3:** GPs’ evaluation of SPHC importance (index), univariate analyses

Variable	Regression Coefficient β	Significance	95% Confidence Interval	Standard Error	n
Age	−.002	.238	−.006; .001	.002	954
Gender (Reference: male)	.158	<.001	.093; .223	.033	955
Work experience (years)	−.003	.023	−.006; −4.8*10^(−4)	.002	948
Working hours/week	−.005	<.001	−.008; −.002	.001	944
Practice Type (Reference: medical care centre)					956
Single-handed	−.123	.128	−.281; .035	.081	
Group practice	−.123	.128	−.282; .035	.081	
Employed (reference: yes)	−.064	.218	−.165; .038	.052	943
Location of practice (reference: big city)					949
Rural	−.084	.070	−.176; .007	.047	
Small town	−.057	.212	−.147; .033	.046	
Medium-sized town	−.078	.124	−.177; .021	.050	
Affiliation to Federal State (association of Statuatory Health Insurance Physicians, reference: Westphalia-Lippe)					956
Bavaria	.039	.561	−.093; .171	.067	
Berlin	.227	.003	.076; .378	.077	
Hesse	.144	.040	.006; .282	.070	
Lower Saxony	.108	.133	−.033; .248	.072	
Saxony-Anhalt	.068	.313	−.064; .200	.067	
Schleswig-Holstein	.083	.235	−.054; .220	.070	
Thuringia	.122	.056	−.003; .247	.064	
Number of patients/quarter	−9.08*10^(−5)	.048	−1.81*10^(−4)	−7.68*10^(−7)	910
Number of palliative patients/year	−.001	.081	−.002; 1.40*10^(− 4)	.001	940
Number of home visits/week	−.001	.096	−.003; 2.42*10^(− 4)	.001	954
Number of home visits/quarter within palliative patients	−3.27*10^(−4)	.051	−.001; 1.46*10^(−6)	1.67*10^(− 4)	937
Conviction that palliative care should be a central part of GP’s work	−.145	<.001	−.185; −.106	.020	949
Self-assessed palliative competence	−.182	<.001	−.230; −.135	.024	939
Extent of GP palliative care delivery (index)	−.334	<.001	−.395; −.272	.031	954
Perceived involvement in treatment after SPHC initiation	.116	<.001	.080; .152	.018	836
Frequency of SPHC referrals denied by MDK^a^	.017	.617	−.049; .082	.033	811
Number of SPHC referrals by other health care professionals	−.007	.035	−.014; −.001	.003	756
Qualification level (Reference: none + exclusively within work in general practice)					921
Additional qualification in palliative care	−.080	.158	−.191; .031	.057	
BQKPmV^b^	−.337	.084	−.720; .045	.195	
40 h-course certificate	−.068	.154	−.161; .025	.047	
Having worked in a palliative care institution for at least 3 months	.069	.342	−.073; .210	.072	
Remuneration level (Reference: PPC)					956
Settlement via selective contracts	−.087	.168	−.212; .037	.063	
BQKPmV	−.399	.008	−.693; −.105	.150	
PPC + additional qualification in palliative care	−.081	.201	−.204; .043	.063	
Quality of surrounding palliative infrastructure	.121	.003	.042; .201	.041	869
Quality of utilised SPHC	.241	<.001	.187; .294	.027	811

^a^ Medical advisary service of Social Health Insurance (MDK: Medizinischer Dienst der Krankenkassen)

^b^ Particularly qualified and coordinated general palliative care (translation)

Inversely associated with GP-valued SPHC importance were: a certain remuneration level linked to specific palliative care qualification requirements, extent of reported GP palliative care delivery, self-assessed competence, and the conviction that palliative care should be a central part of GPs’ work. Furthermore, the frequency of SPHC referrals through other providers, perceived workload, duration of work in the outpatient sector and patients per quarter were related to a lower SPHC importance index. Noticeable differences were found between federal states.

Twenty-two explanatory variables (Table 3) were included in the final multiple regression. A residual analysis confirmed that the conditions for multiple regression were met. After all variables were simultaneously entered into the model, four variables finally remained statistically significant (explaining 18.3% of the total variance, R^2^) showing a significant correlation with GP perceived SPHC importance (Table 4): the reported extent of GPs’ palliative care delivery, the perceived quality of the utilised SPHC services, the conviction that palliative care should be a central part of GPs’ work and involvement in the treatment of palliative patients after SPHC initiation.

**Table 4:** GPs’ evaluation of SPHC importance (index), multiple regression analysis^a^

Variable	Regression Coefficient β	Signifi-cance	95% Confidence Interval	Standard Error
(Constant)	2.30	<.001	1.69; 2.92	.315
Extent of GP palliative care delivery (index)	−.283	<.001	−.384; −.182	.051
Conviction that palliative care should be a central part of GPs' work	−.062	.025	−.116; −.008	.027
Perceived involvement in treatment of palliative patients after SPHC initiation	.088	<.001	.042; .134	.023
Quality of utilised SPHC	.119	.001	.048; .190	.036

^a^ corrected R^2^ = .183, *n* = 559; only independent variables with significance < 0.05 are shown

Additional univariate  analysis demonstrated that the SPHC importance index is solidly associated with the reported number of SPHC referrals (*n* = 808; β = 0.138; *p* < 0.001) displaying that GPs with low own palliative care activity and high perception of SPHC-importance reported more SPHC referrals, and GPs with high own palliative care activity and low perception of SPHC-importance reported less SPHC referrals.

## Discussion

### Main finding

The primary finding of this study was that GPs’ perception of SPHC importance (as well as reported initiation of SPHC) is mainly driven by their own reported extent of palliative care activities: a GP with high perceived palliative activity values SPHC less; a GP with low activity values SPHC more.

To our knowledge, this is the first (quantitative) study revealing this interrelation from which specific conclusions can be drawn.

The single palliative care activity for which GPs most frequently deemed SPHC support beneficial was “technical and invasive treatment measures”, such as morphine pumps, subcutaneous infusions and port systems, required to ensure sufficient symptom control. It is known that the wish to relieve patients’ suffering [16], the non-availability of equipment [17] and low self-confidence in applying those measures [18] might lead GPs to seek support, especially with regard to therapeutic options [16, 19]. Beyond that, specialist support in “treatment in the final phase” was attributed high importance. As patients deteriorate, they are often in need of intensified therapies [20] and increased frequency of out-of-hours home visits [21] – requirements that overload GPs. Additionally, GPs value SPHC more during out-of-hours times (holidays, weekends). This is in line with previous findings which have identified GPs’ lack of time and inadequate resources as significant barriers [22–24] to continuously providing palliative care in the final phase.

### Further findings

Further factors influencing GPs' perception of SPHC importance: Firstly, the higher GPs’ perceived SPHC quality the higher they rated the importance of SPHC. Although there is no uniform definition of quality of SPHC, recent studies describe factors contributing to GPs’ satisfaction with SPHC teams: timely and appropriate advice and accessible help [25–27], well-developed channels of communication, timely provision of technical expertise [8] and competence of health care providers [27].

Secondly, if SPHC had been initiated, the continuing involvement in the treatment of their patients appears to be relevant to GPs, resulting in a higher appreciation of SPHC. GPs’ wish to stay involved in their patients’ treatment even if SPHC is involved has been repeatedly emphasised [28]. More critical GP voices regard SPHC as constantly replacing their own role by reducing the active part of GPs in palliative care delivery to bureaucratic functions (i.e. medication prescriptions) [29]. A similar sentiment is displayed by the finding that SPHC referrals by other health care providers (e.g. hospitals) lead to a lack of consideration of the GP’s role [30], impeding their perception of SPHC importance.

### Interpretation

The context of current primary care in Germany reveals a highly pressurised work environment [31] with a high workload, which is even more pronounced in rural areas. Hence, the engagement of GPs in palliative care might be impeded due to a lack of resources [24, 32] and limited capacities (experience, competence, knowledge) [33]. Moreover, intergenerational changes in the basic attitude towards the GP profession may lead to decreasing readiness to provide 24 h-care particularly in younger GPs. This is supported by the finding that the extent of GPs’ palliative care activities rises with age [34].

Nonetheless, a surrounding palliative care infrastructure besides from SPHC (nursing care services, inpatient palliative care, hospices) GPs perceive as insufficient, keeps them from taking over own responsibility for end-of-life care and drives them to involve SPHC [34] (data analysed, not published yet). As the palliative infrastructure strongly varies between regions [35], GPs’ palliative care activities also vary between regions as then does the involvement of SPHC.

As the study design did not capture information on the patient, it remains uncertain how GPs identified a patient’s medical need for SPHC. But as it is known that GPs’ palliative care activities increase with experience [16], competence [36] and training [37]), we principally assume that GPs’ ability to assess the medical need of SPHC improves with the extent of their engagement in palliative care. Against this background, we interpret our results that GPs who are not greatly engaged in palliative care activities involve SPHC as a substitution for PPC tasks they cannot or are not willing to provide themselves due to a lack of resources. Thus, SPHC is partially doing tasks that could be done within PPC if GPs had the necessary resources (time, confidence, professional and financial support). The interpretation is in line with a previous qualitative study by Schneider et al. [29] who already pointed out the link between lacking or insufficient PPC resources and inappropriate SPHC-involvement.

### Implications for palliative care politics

Firstly, instead of reinforcing GPs’ withdrawal from palliative care delivery by shifting traditional tasks from GPs to specialists [30], strengthening PPC could be promising. Here, our survey confirms starting points that have been found in qualitative study designs already: Primary care professionals have the potential, ability and motivation to provide palliative care for most patients [1, 38] given adequate training, resources, remuneration, a supportive and cooperative surrounding palliative care infrastructure [34] and specialist advice when needed [24, 38, 39]. The latter is particularly important for training GPs in identifying complex cases [40] and for guaranteeing highly qualitative primary palliative care provision during out-of-hours [40, 41].

Nevertheless, there are limits to an enforcement of PPC, due to the high workload GPs in Germany face, which is particularly pronounced in rural areas. That is why, secondly, it should be acknowledged that partially SPHC fills gaps in PPC. This appears to be particularly relevant for 24 h-availability, support in technical care and in the final phase – activities that do not necessarily require special qualifications. And, as there is large regional variation in the capacity of PPC and the general palliative care infrastructure, SPHC has to be regarded as an additional palliative care resource and has to adapt flexible to regional PPC structures.

However, to fill gaps in PPC and to support PPC efficiently, SPHC has to be adjusted with appropriate remuneration structures and adequate – which means not too high – minimum standards for qualification requirements for SPHC-teams as well as requirements regarding a minimum number of professions involved in SPHC. In addition, new forms of cooperation between PPC and SPHC can arise, should be tested and fostered. But always, outcomes of PPC and SPHC (like patient/relatives’ satisfaction as well as costs of care) have to be taken into account when setting the framework.

Not to forget, thirdly, to reduce patients’ and relatives’ need for palliative support via improving self-help which will take burden from PPC as well as SPHC.

These are all future tasks of palliative (home) care politics our results are pointing at.

### Strength and limitations

This is the first comprehensive, nationwide survey among German GPs addressing the interface of general and specialised palliative homecare from their perspective.

Even though the response rate of 19.4% was not too low a response rate compared to our expectations, it naturally limits the representativeness of our results.

Anticipating that responding GPs might be more enthusiastic towards participation in palliative care (apparent in the overrepresentation of GPs with an additional qualification in palliative care), we considered formal experience/qualification level (Additional file 1) as an independent variable in our analysis to avoid a significant distortion in the results attained. Furthermore, we had excluded SPHC-GPs beforehand (*n* = 118) as we supposed that there would be selection bias due to a conflict of interest, especially in terms of remuneration. Only in the descriptive results does the overrepresentation of GPs with higher palliative care experience/qualification remain.

There may be limitations in terms of the transferability of results to other high-income countries due to health system differences and differences in palliative care delivery. But the general observation of GP’ (reported) palliative care activities being inversely associated with GP-valued SPHC importance, the interpretation of this interrelation and the conclusion for actions drawn from this, might, at least partly, also hold for countries other than Germany.

## Conclusions

GPs with low reported activity in palliative care are more likely to initialise SPHC for palliative care activities they do not deliver themselves for various reasons, which might mean that the involvement of SPHC is substitutive instead of complementary to primary palliative care. This finding and its interpretation should be given more attention in the future policy framework for (specialised) palliative homecare in Germany.

## Supplementary information

**Additional file 1.** The development of the questionnaire.

**Additional file 2.** Questionnaire: English version.

**Additional file 3.** GP perceived valuing of SPHC activities.

## Data Availability

Additional file 2 contains the questionnaire. The datasets used and/or analysed during the current study are available from the corresponding author on reasonable request.

## Abbreviations

PPC: Primary palliative care, which means generalist palliative homecare (German abbreviation: AAPV)
BQKPmV: Particularly qualified and coordinated general palliative care (translation)
G-BA: Federal Joint Committee, the highest decision-making body of the joint self-government of physicians, dentists, hospitals and health insurance funds in Germany
KV: (regional) Association of Statutory Health Insurance Physicians
SPHC: Specialised palliative homecare (German abbreviation: SAPV)
SGB: Social Code Book
