# Supplementary material for: Factors influencing GPs’ perception of specialised palliative homecare (SPHC) importance – results of a cross-sectional study
Source: BMC Palliat Care. 2020 Aug 3;19:117. doi: 10.1186/s12904-020-00603-3 (PMC7401213; doi:10.1186/s12904-020-00603-3)
Supplement: Supplementary file 1 — Additional file 1. The development of the questionnaire. [file 12904_2020_603_MOESM1_ESM.docx]

**Supplementary material to the original article from Stichling et al. „Factors influencing GPs’ perception of specialised palliative homecare (SPHC) importance – results of a cross-sectional study”**

Additional file 1: The development of the questionnaire

The development of the questionnaire was based on a thorough literature search and semi-structured and protocolled interviews with four GPs in different interfaces to SPHC. For ensuring that all important aspects were covered and comprehensive, the questionnaire was discussed in two multidisciplinary and inter-professional conferences which resulted in slight changes of structure and linguistic issues. A pilot study involving GPs from Thuringia (n=7), Berlin (n=1) and Westphalia-Lippe (n=2) was conducted to test its applicability in different federal states due to heterogeneous organizational structures. As no problem was detected the number of GPs included in pilot testing was not increased.
