# Supplementary material for: Factors influencing GPs’ perception of specialised palliative homecare (SPHC) importance – results of a cross-sectional study
Source: BMC Palliat Care. 2020 Aug 3;19:117. doi: 10.1186/s12904-020-00603-3 (PMC7401213; doi:10.1186/s12904-020-00603-3)
Supplement: Supplementary file 2 — Additional file 2. Questionnaire: English version. [file 12904_2020_603_MOESM2_ESM.pdf]

GP survey on palliative care activity to gain insight into the interrelation of PPC and SPHC

Advice for filling in the questionnaire:

- Only use a pen (no pencil).
- Clearly place a mark in the box of your answer: X
- In case of a falsely marked box, fill in the box completely ■ and mark your right choice again: X

A01	How old are you?	☐
A02	Select your gender:	☐ female ☐ male
A03	For how long have you been working as a physician in the outpatient sector (in years)?	☐ years (incl. further medical education)
A04	Are you working in a single practice or in a collaborative form?	☐1 single-handed practice<br>☐2 group practice<br>☐3 medical care centre
A05	Are you working as an employee?	☐ yes ☐ no
A06	To which Association of Statutory Health Insurance Physicians (KV) do you belong?	☐1 Bavaria<br>☐2 Berlin<br>☐3 Hesse<br>☐4 Lower-Saxony<br>☐5 Saxony-Anhalt<br>☐6 Schleswig-Holstein<br>☐7 Thuringia<br>☐8 Westfalia Lippe
A07	In which region is your practice located?	☐1 Rural (≤ 5,000 inhabitants)<br>☐2 Small town (> 5,000 – 20,000 inhabitants)<br>☐3 Medium-sized Town (> 20,000 – 100,000 inhabitants)<br>☐4 Big city (> 100,000 inhabitants)
A08	How many hours do you work on average per week? (approximately)	☐
A09	How many patients do you have on average per quarter? (approximately)	☐
A10	How many home visits do you perform per week? (approximately)	☐
A11	How many palliative patients do you have per year? (approximately)	☐
A12	How many home visits do you perform for palliative patients in a quarter? (approximately)	☐

A13	**Contact with palliative care:** In which ways have you had contact with palliative care during your medical practice so far? (Multiple selection possible)
	☐ _1 none<br>☐ _2 previously working in a palliative care institution for at least 3 months<br>☐ _3 40-hour course certificate<br>☐ _4 additional qualification in palliative care<br>☐ _5 BQKPmV- Physician<br>☐ _6 current participation in a specialised palliative homecare-Team (SPHC)<br>☐ _7 settlement via selective contracts for palliative care (§73b/140a SGB V, i.e. integrated care, palliative networks, etc.)<br>☐ _8 exclusively within the work in general practice

	**How much do you agree with each of the following statements? Please mark your answer.**<br><br>**The care of seriously ill and dying patients…**	**strongly disagree**			**strongly agree**
B01	…should be a central part of GPs' work.	☐	☐	☐	☐
B02	…is considerably facilitated by the involvement of caregivers.	☐	☐	☐	☐
B03	…should be coordinated by the general practitioner.	☐	☐	☐	☐
B04	...should be a multi-professional and interdisciplinary field.	☐	☐	☐	☐
B05	...should be continuously supported by GPs until death.	☐	☐	☐	☐
B06	…should be a field of palliative specialists (except GPs).	☐	☐	☐	☐

	**As a general practitioner, I feel secure in …**	**never**			**always**
C01	…identifying palliative patients.	☐	☐	☐	☐
C02	…adapting the therapy to the palliative situation of the patients.	☐	☐	☐	☐
C03	…starting to talk with patients about their poor prognosis.	☐	☐	☐	☐
C04	…dealing with "difficult" relatives of patients.	☐	☐	☐	☐
C05	…reducing/ending therapies in the final phase.	☐	☐	☐	☐
C06	…performing technical treatment measures (e.g. flushing of port systems).	☐	☐	☐	☐
C07	…working together with other health care professionals (SPHC team, etc.).	☐	☐	☐	☐
C08	…accepting and supporting the preferences of the patients concerning therapeutic decisions.	☐	☐	☐	☐
C09	…timely handing over of the patient to specialised palliative care structures when a transgression of competence threatens.	☐	☐	☐	☐
C10	…withstand emotional and stressful situations.	☐	☐	☐	☐
C11	**Please assess your overall palliative competence/expertise.**	**bad**<br>☐	☐	☐	**good**<br>☐

	Palliative infrastructure: Is the healthcare provider below available in the setting of your office? If yes, please assess the quality of the care.	Availability_1			If available and used at least once, assess the quality of care.2			
		unknown	yes	no	bad			good
D01	own, non-medical employee for palliative medical home visits (“VERAH”/”NÄPA”)		☐	☐	O	O	O	O
D02	nursing care service	☐	☐	☐	O	O	O	O
D03	specialised palliative nursing care service	☐	☐	☐	O	O	O	O
D04	further services (outpatient hospice, “Grüne Damen”, caritative visit)	☐	☐	☐	O	O	O	O
D05	outpatient PKD (palliative care service)	☐	☐	☐	O	O	O	O
D06	specialist with additional qualification in palliative care (qualified palliative physician)	☐	☐	☐	O	O	O	O
D07	(other) physician with BQKPmV-qualification	☐	☐	☐	O	O	O	O
D08	SPHC-team	☐	☐	☐	O	O	O	O
D09	palliative care unit	☐	☐	☐	O	O	O	O
D10	inpatient hospice	☐	☐	☐	O	O	O	O
D11	homecare companies (caregiver for adjuvants, rehabilitation and pharmaceuticals)	☐	☐	☐	O	O	O	O
D12	**Please assess the overall quality of care of the surrounding palliative infrastructure.**				bad ☐	☐	☐	good ☐

E01	**Have you ever referred to SPHC in your work as a general practitioner? Please mark your answer.**	☐ yes ☐ no<br>**(If no, please skip to page 4)**
E02	How many referrals of SPHC do you make per year (including follow-up referrals)?	☐
E03	How many of your patients get a referral to SPHC from other healthcare providers (hospital/medical specialists) per year?	☐
E04	Do you stay involved in the treatment of your patients after initialising SPHC?	never ☐ ☐ ☐ ☐ always
E05	How often do health insurance services reject your referral to SPHC?	☐ ☐ ☐ ☐

	**Which service do you usually refer to according to form no. 63 ("Muster 63")?** **Please mark your answer.** (Multiple selection possible)	
	Consulting	
F01	a) of the treating physician	☐
F02	b) of the treating nurse	☐
F03	c) of the patients/relatives	☐
F04	Coordination of palliative care	☐
F05	(additional) partial service	☐
F06	full service	☐

	**Which characteristics does the SPHC team you usually work with have?** **Please mark your answer.**	**unknown**	**yes**	**no**
G01	SPHC team offers full service	☐	☐	☐
G02	sufficient admission capacity	☐	☐	☐
G03	employs different professions in the team (psychologists, social worker, chaplain, etc.)	☐	☐	☐
G04	good material equipment (e.g. analgesics pumps)	☐	☐	☐
G05	availability for telephone advice at any time	☐	☐	☐
G06	regular meetings for case reports with all healthcare professionals	☐	☐	☐
G07	further training opportunities for cooperating colleagues	☐	☐	☐
G08	medical direction of the SPHC-Team through…	☐		
G09	…hospital doctors		☐	
G10	…general practitioners		☐	
G11	…outpatient specialists (anaesthetists, oncologist, etc.)		☐	
G12	settlement of the SPHC-Team…	☐		
G13	…at a single practice or practice network		☐	
G14	…at a hospital		☐	
G15	…at a hospice or outpatient hospice service		☐	
G16	…at a nursing care service		☐	
G17	…at other institutions (palliative base, network, etc.)		☐	

	Palliative care involves a wide spectrum of activities. How often do you take responsibility for the following activities for your palliative patients in their homes?[1]					For this, how important is an SPHC team/PKD for you?[2]		
	**Assessment and care plan:**	never			always	not required	important	very important
H01	perform starting assessment	☐	☐	☐	☐	○	○	○
H02	identify that SPHC is needed	☐	☐	☐	☐	○	○	○
H03	give support in the context of patient decree and power of attorney	☐	☐	☐	☐	○	○	○
H04	develop a treatment and emergency plan	☐	☐	☐	☐	○	○	○
H05	counsel and guide patients and relatives	☐	☐	☐	☐	○	○	○
H06	document/re-evaluate treatment measures	☐	☐	☐	☐	○	○	○
**Symptom management:**								
H07	pain	☐	☐	☐	☐	○	○	○
H08	dyspnoea	☐	☐	☐	☐	○	○	○
H09	nausea/vomiting, lack of appetite, obstipation	☐	☐	☐	☐	○	○	○
H10	fatigue, depression, anxiety, delirium	☐	☐	☐	☐	○	○	○
**Interventions:**								
H11	identify needs and prescribe pharmaceuticals and devices	☐	☐	☐	☐	○	○	○
H12	prescribe medication for pain and palliative symptoms	☐	☐	☐	☐	○	○	○
H13	prescribe medication, pharmaceuticals and devices which heavily burden my budget	☐	☐	☐	☐	○	○	○
H14	identify needs and prescribe home care	☐	☐	☐	☐	○	○	○
H15	decide on involving nursing care services	☐	☐	☐	☐	○	○	○
H16	treat comorbidities/chronic diseases	☐	☐	☐	☐	○	○	○
H17	palliative medical wound care	☐	☐	☐	☐	○	○	○
H18	artificial nutrition and replacement of fluids	☐	☐	☐	☐	○	○	○
H19	technical and invasive treatment measures	☐	☐	☐	☐	○	○	○
H20	treatment in the final phase	☐	☐	☐	☐	○	○	○
H21	reduce/end therapies	☐	☐	☐	☐	○	○	○
**Coordination:**								
H22	coordinate all diagnostic, therapeutic and nursing measures, and caregivers	☐	☐	☐	☐	○	○	○
H23	psychosocial care	☐	☐	☐	☐	○	○	○
H24	involve outpatient hospice service	☐	☐	☐	☐	○	○	○
H25	organise specialist visits, transports, and admissions	☐	☐	☐	☐	○	○	○
H26	(interdisciplinary) case conferences with other involved professionals	☐	☐	☐	☐	○	○	○
H27	perform councils	☐	☐	☐	☐	○	○	○
**Availability:**								
H28	home visits by myself	☐	☐	☐	☐	○	○	○
H29	home visits by own non-medical practice employee	☐	☐	☐	☐	○	○	○
H30	be available for visits during the opening hours	☐	☐	☐	☐	○	○	○
H31	be available for visits outside the opening hours (incl. weekends/holidays/at night)	☐	☐	☐	☐	○	○	○
H32	(only) telephone advice outside the opening hours	☐	☐	☐	☐	○	○	○

	Which conditions would facilitate the palliative care of your patients? Please mark your answer.	Don't know	strongly disagree			strongly agree
J01	possibilities for immediate advice in palliative questions	☐	☐	☐	☐	☐
J02	remuneration according to expenses (home visits, counselling by phone, 24-hour care, etc.)	☐	☐	☐	☐	☐
J03	available (interdisciplinary) trainings for all healthcare providers	☐	☐	☐	☐	☐
J04	possibility for care coordination and transition management by special trained staff (Case Manager)	☐	☐	☐	☐	☐
J05	involving bereavement care within the palliative care concept	☐	☐	☐	☐	☐
J06	standardised exchange of information with all involved healthcare professionals	☐	☐	☐	☐	☐
J07	reinforcement of the hospice culture	☐	☐	☐	☐	☐
J08	better perception and coverage of palliative patients' needs in nursing homes	☐	☐	☐	☐	☐
J09	better palliative qualification of outpatient nursing care services	☐	☐	☐	☐	☐
J10	reinforcement of volunteer work in palliative care	☐	☐	☐	☐	☐
J11	establishing "Advanced Care Planning" in palliative care	☐	☐	☐	☐	☐
J12	building the capacity of SPHC	☐	☐	☐	☐	☐
J13	others, namely:<br>…………………………………………<br>…………………………………………<br>…………………………………………<br>…………………………………………<br>…………………………………………					

Thank you for your assistance!
