# Supplementary material for: Factors influencing GPs’ perception of specialised palliative homecare (SPHC) importance – results of a cross-sectional study
Source: BMC Palliat Care. 2020 Aug 3;19:117. doi: 10.1186/s12904-020-00603-3 (PMC7401213; doi:10.1186/s12904-020-00603-3)
Supplement: Supplementary file 3 — Additional file 3. GP perceived valuing of SPHC activities. [file 12904_2020_603_MOESM3_ESM.docx]

**Supplementary material to the original article from Stichling et al. „Factors influencing GPs’ perception of specialised palliative homecare (SPHC) importance – results of a cross-sectional study”**

Additional file 3: GP perceived valuing of specialised palliative homecare (SPHC) activities

|  | N  904  907  915  897  880  888 |
| --- | --- |
|  | N  895  917  921  916 |
|  | N  913  924  922  924  919  911  914  912  917  916  917 |
|  | N  889  897  896  885  905  909 |
|  | N  894  900  871  836  862 |
